# Epidemiology and associated factors of polypharmacy in older patients in primary care: a northern Italian cross-sectional study

**DOI:** 10.1186/s12877-021-02141-w

**Published:** 2021-03-20

**Authors:** Giuliano Piccoliori, Angelika Mahlknecht, Marco Sandri, Martina Valentini, Anna Vögele, Sara Schmid, Felix Deflorian, Adolf Engl, Andreas Sönnichsen, Christian Wiedermann

**Affiliations:** 1 Institute of General Practice and Public Health, College of Health Care Professions, Lorenz Böhler- Straße 13, 39100 Bolzano, Italy; 2 South Tyrolean Academy of General Practice, Wangergasse 18, 39100 Bolzano, Italy; 3 Institute of General Practice, Family Medicine and Preventive Medicine, Paracelsus Medical University, Strubergasse 21, 5020 Salzburg, Austria; 4 Big & Open Data Innovation Laboratory (BODaI-Lab), University of Brescia, Via S. Faustino 74/B, 25122 Brescia, Italy; 5 Department of General Practice and Family Medicine, Center for Public Health, Medical University of Vienna, Kinderspitalgasse 15/I, 1090 Vienna, Austria; 6 UMIT – Private University for Health Sciences, Medical Informatics and Technology – Tyrol, Eduard-Wallnöfer-Zentrum 1, 6060 Hall in Tirol, Austria

**Keywords:** Polypharmacy, Inappropriate prescribing, Older adults, General practice, Drug interactions

## Abstract

**Background:**

A precondition for developing strategies to reduce polypharmacy and its well-known harmful consequences is to study its epidemiology and associated factors. The objective of this study was to analyse the prevalence of polypharmacy (defined as ≥8 prescribed drugs), of potentially inappropriate medications (PIMs) and major drug-drug interactions (DDIs) among community-dwelling general practice patients aged ≥75 years and to identify characteristics being associated with polypharmacy.

**Methods:**

This cross-sectional study is derived from baseline data (patients’ demographic/biometric characteristics, diagnoses, medication-related data, cognitive/affective status, quality of life) of a northern-Italian cluster-RCT. PIMs and DDIs were assessed using the 2012 Beers criteria and the Lexi-Interact® database. Data were analysed using descriptive methods, Wilcoxon rank-sum tests, Fisher’s exact tests and Spearman correlations.

**Results:**

Of the eligible patients aged 75+, 13.4% were on therapy with ≥8 drugs. Forty-three general practitioners and 579 patients participated in the study. Forty five point nine percent of patients were treated with ≥1 Beers-listed drugs. The most frequent PIMs were benzodiazepines/hypnotics (19.7% of patients) and NSAIDs (6.6%). Sixty seven point five percent of patients were exposed to ≥1 major DDI, 35.2% to ≥2 major DDIs. Antithrombotic/anticoagulant medications (30.4%) and antidepressants/antipsychotics (23.1%) were the most frequently interacting drugs. Polypharmacy was significantly associated with a higher number of major DDIs (Spearman’s rho 0.33, *p* < 0.001) and chronic conditions (Spearman’s rho 0.20, *p* < 0.001), higher 5-GDS scores (thus, lower affective status) (Spearman’s rho 0.12, *p* = 0.003) and lower EQ-5D-5L scores (thus, lower quality of life) (Spearman’s rho − 0.14, *p* = 0.001). Patients’ age/sex, 6-CIT scores (cognitive status), BMI or PIM use were not correlated with the number of drugs.

**Conclusions:**

The prevalence of polypharmacy, PIMs and major DDIs was considerable. Results indicate that physicians should particularly observe their patients with multiple conditions, reduced health and affective status, independently from other patients’ characteristics. Careful attention about indication, benefit and potential risk should be paid especially to patients on therapy with specific drug classes identified as potentially inappropriate or prone to major DDIs in older persons (e.g., benzodiazepines, NSAIDs, protonic pump inhibitors, antithrombotics/anticoagulants, antidepressants/antipsychotics).

**Trial registration:**

The cluster-RCT on which this cross-sectional analysis is based was registered with Current Controlled Trials Ltd. (ID ISRCTN: 38449870) on 2013-09-11.

## Background

In Europe, chronic conditions are the leading cause of illness and disability and constitute large parts of healthcare costs [1]. Especially older-aged persons are not only likely to suffer from chronic conditions, but also to be affected from *multiple* diseases [2] with the consequence of reduced quality of life and impaired health outcomes. The phenomenon of multi-morbidity is highly prevalent in the older-aged population and raises complex needs of care as each condition can influence the clinical and therapeutic course of other concomitant pathologies [3]. This renders drug therapy challenging and entails the risk for polypharmacy.

A general consensus regarding the cut-off point defined as polypharmacy does not exist. In primary care, the most common cut-off is a use of ≥5 drugs [4]. Depending on definition and setting, up to 54% of older persons have shown to be affected from polypharmacy [5]. This number rises up to 79% in residential care [6]. A cross-sectional analysis including 17 European countries and Israel found a polypharmacy rate of 26.3-39.9% among persons aged 65+. The highest prevalence of polypharmacy was found in Portugal, Israel and the Czech Republic, whereas Switzerland, Croatia and Slovenia were the countries with the lowest polypharmacy rates [7].

In Germany, the age group 60+ was found to receive two thirds of all prescribed drugs [8]. In Italy, about two thirds of persons aged 65+ were prescribed four or more active agents per year and the older-aged population has shown to absorb nearly 2/3 of the public pharmaceutical expenditure [3, 9].

Besides from a notable economic impact, polypharmacy has shown to entail several clinically harmful effects: increased risk for potentially inappropriate medications (PIMs) [10], under-use of appropriate medications, low patient compliance, drug-drug interactions (DDIs) [3], adverse drug events (ADEs) [11], functional decline [12], lower physical performance [13], hospitalisations due to ADEs (predictable from the known pharmacology of the prescribed drugs e.g. interactions, and therefore avoidable in 59-70%) [14], short-time hospital readmissions [15], and even increased mortality [14].

Thus, polypharmacy has become a relevant public health issue and a major concern regarding patient safety in the field of medical treatment of older people.

The proportion of persons aged 65 or more years is increasing in the European population, especially the proportion of the oldest old (80 years or more) [16]. In the UK, one in 12 persons is estimated to be aged 80 or more years by 2039 [17]. In Italy, persons aged ≥65 years are estimated to account for 33% of the general population in 2051 [3]. At the same time, as drug use in older-aged patients is common, the consumption of drugs and the pharmaceutical expenditures are rising in Italy as well as in other countries [3].

Thus, polypharmacy will be of increasing clinical significance especially in general practice as the GPs are the major initiators and providers of drug prescriptions: although exact figures are not available, it may be assumed that 60-80% of prescriptions are initiated by GPs [18].

It is, therefore, crucial to study the epidemiology of polypharmacy and its associated factors in older-aged persons as a precondition for developing strategies to reduce polypharmacy and potentially harmful consequences in general practice. In the inpatient setting, efforts in this regard have been conducted since several years, e.g. the prospective REPOSI register which was started in 2008 with the aim to study the prevalence of polypharmacy and to improve medication appropriateness for older persons in Italy [9]; in European general practice, up to now, only few studies investigating the prevalence and predictors of polypharmacy have been conducted [19].

We therefore initiated the cluster-randomised controlled trial (RCT) ‘PRIMA’ (**P**olypharmacy in chronic diseases **- R**eduction of **I**nappropriate **M**edication and **A**dverse drug events in older populations) (2014-2016) with the objective to investigate the impact of an intervention aiming at reducing polypharmacy on mortality/hospitalisation.^1^

In this article, we present the cross-sectional analysis of the epidemiological baseline data of the RCT with the aim

- ▪ To analyse the prevalence of polypharmacy (defined as ≥8 prescribed drugs), PIMs and DDIs among community-dwelling general practice patients aged ≥75 years in a northern Italian region
- ▪ To detect associated factors with polypharmacy in general practice.

The cut-off of ≥8 drugs was chosen for the underlying cluster-RCT as the sample-size calculation was based on a previous study [20] where the participating patients were treated on average with a corresponding number of medications.

## Methods

### Study design, setting and population

The cross-sectional study was conducted in the primary care setting in the province of Bolzano (Italy) and involved GPs and older-aged community-living patients.

### Recruitment

All 270 active GPs listed in the Chamber of Physicians of Bolzano were informed and invited by email and phone to participate.

The participating GPs identified eligible patients meeting the following inclusion criteria:

- Age ≥ 75 years
- On therapy with ≥8 prescribed active agents (excluding PRN-medications and OTC-drugs)
- Absence of terminal illness/radiation/chemotherapy
- Sufficient cognitive function to be able to give informed consent.

The patients were consecutively invited to participate by the GPs during routine visits in the GP office. All participating GPs and patients gave written informed consent.

### Data collection

Data were collected by means of structured case report forms (CRFs). For the *n* = 39 GPs using the electronic health record (EHR) Millewin®, an add-on module was programmed which filled in automatically all required patient data which were available in the EHR. The GPs checked these electronically generated CRFs and completed all missing data manually (e.g., the results of the questionnaires, see below).

The following parameters were collected:

- ▪ Patientʼs *demographic data*: age, sex
- ▪ Patientʼs *diagnoses* (ICD-9-coded)
- ▪ Current *medication* (International Non-proprietary Names) and daily dosage in milligrams
- ▪ *Biometric and laboratory parameters*: height, weight, BMI, blood pressure, creatinine, potassium
- ▪ *Health-related quality of life, cognitive function* and *affective status* were measured by the EQ-5D-5L [21], the 6-Item Cognitive Impairment Test (6-CIT) [22], and the 5-Item Geriatric Depression Scale (5-GDS) [23]. The questionnaires were handed out to the patients by the GPs who recorded the results in the case report forms.

Data were pseudonymised by the GPs. Afterwards, the electronic CRFs were forwarded by email to the research team via the add-on module.

### Data analysis

The drug regimens of all participating patients were assessed by two members of the project team (one GP, not included as study participant, and one student of pharmacology) regarding *PIMs* using the 2012 Beers criteria (Italian Version) [24, 25] and *DDIs* using the Lexi-Interact® database [26]. Only potentially severe DDIs were considered: categories D = consider drug modification, and X = avoid combination.

Values obtained from EQ-5D-5L were converted into the *EQ-5D index* (single value per patient; maximum = 1 = full health) by using the German EQ-5D-5L Crosswalk Value Set [27] as no country-specific value set was available for Italy [28] and Germany most closely approximates to the investigated northern Italian region^2^ [29].

*Statistical analysis* was performed by an independent statistician via Stata 16.1 (StataCorp. 2019. Stata Statistical Software: College Station, TX). Categorical variables were summarised as absolute and relative frequencies, while numerical variables as median and interquartile range (IQR), as appropriate.

Wilcoxon rank-sum test and Fisher’s exact test were used to compare the distribution of continuous and categorical variables between study groups, respectively. Spearman correlations were used to assess associations between variables (crude and adjusted for age, gender, number of chronic conditions, cognitive function) [30]. Moreover, logistic regression models were applied for the estimation of unadjusted and adjusted odds ratios (ORs) as a measure of the association between hyperpolypharmacy (defined as the use of ≥10 drugs) and various study variables [12, 13]. All tests were two-sided, the level of statistical significance was set at *p* < 0.05.

*Missing data*: Demographical data of GPs/patients, diagnoses and medication-related data were complete. Laboratory values were not available for all patients; in case of missing values, a listwise deletion was applied (individuals with missing data were excluded from analysis of laboratory values).

## Results

### Study participants, conditions and drug use

Of 270 invited GPs, 43 (15.9%) participated in the study.

The participating GPs treated 71,014 patients overall and 8015 patients aged ≥75 years. Of these, 1075 patients (13.4%) were on regular therapy with ≥8 drugs and thus eligible. The percentage of patients on polypharmacy (on therapy with ≥8 drugs) among those aged 75+ varied between 5 and 37% per GP.

A total of 579 patients (53.9% of the eligible patients) took part in the study. Characteristics of the study participants are summarised in Table 1.

**Table 1:** Characteristics of the participating GPs (*n* = 43) and patients (*n* = 579)

**Participating GPs**		
**Age**		Median (IQR)
		56 (49 – 61)
**Sex**	n GPs	% of participating GPs
Male	31	72.1%
Female	12	27.9%
**Location of GP office**	n GPs	% of participating GPs
Urban area	21	48.8%
Rural area	22	51.2%
**Participating patients**		
**Age**		Median (IQR)
		81 (78 – 85)
**Sex**	n patients	% of participating patients
Male	230	39.7
Female	349	60.3
**Biometric and laboratory parameters**		Median (IQR)
BMI [kg/m^2^]		26.5 (23.8-29.4)
Systolic blood pressure [mmHg]		135 (125-143)
Diastolic blood pressure [mmHg]		77 (70-80)
Creatinine [mg/dl]		1.1 (0.9-1.3)
Potassium [mmol/l]		4.3 (4.0-4.7)
**Health-related quality of life**		Median (IQR)
EQ-5D-5L index		0.813 (0.716-0.909)
EQ-VAS score		60.0 (50.0-80.0)
**Cognitive impairment**	n patients	% of participating patients
6-CIT score ≥ 8 points	158	27.3%
**Affective impairment**	n patients	% of participating patients
5-GDS score ≥ 2 points	170	29.4%

*GPs* General practitioners, *IQR* Interquartile range, *BMI* Body mass index, *EQ-5D* 5-Item questionnaire measuring health-related quality of life, *VAS* Visual analogue scale, *6-CIT* 6-Item Cognitive Impairment Test, *5-GDS* 5-Item Geriatric Depression Scale

The participating patients had a total number of 3143 diagnoses of chronic diseases and were treated overall with 5614 prescriptions. A total of 376 patients (64.9% of the participating patients) had ≥5 diagnoses of chronic conditions, 249 patients (43% of the investigated patients) used ≥10 drugs. The most frequent diagnoses and used drug classes are shown in Table 2.

**Table 2:** Frequencies of chronic conditions and drug use among the participating patients (*n* = 579)

**Chronic conditions**			
**Number of diagnoses (total)**	Median (IQR)	Min	Max
3143	5 (4 – 6)	1	14
**Most frequent diagnoses**	n patients	% of participating patients	
Hypertension	490	84.6%	
Arthrosis	266	45.9%	
Diabetes mellitus II	220	38.0%	
Dyslipidaemia	202	34.9%	
Atrial fibrillation	179	30.9%	
Coronary heart disease	175	30.2%	
Osteoporosis	157	27.1%	
Depression	124	21.4%	
Benign prostatic hypertrophy	110	19.0%	
Gastro-oesophageal reflux disease	98	16.9%	
Chronic heart failure	93	16.1%	
Chronic obstructive pulmonary disease (COPD)	81	14.0%	
Chronic renal failure	73	12.6%	
Hyperuricaemia / gout	65	11.2%	
Hypothyroidism	64	11.1%	
Insomnia	60	10.4%	
Cerebrovascular disease / dementia	58 (54 + 4)	10.0% (9.3% + 0.7%)	
**Drug use**			
**Number of drugs (total)**	Median (IQR)	Min	Max
5614	9 (8 – 11)	8	20
**Number of drugs per patient**	n patients	% of participating patients	
8 – 9 drugs	330	57.0%	
≥ 10 drugs	249	43.0%	
**Most frequently used drug classes per patient**^a^	n patients	% of participating patients	
ARBs + ACE-inhibitors	481 (251 + 230)	83.1% (43.4% + 39.7%)	
PPIs	320	55.3%	
Statins	319	55.1%	
Platelet-aggregation inhibitors	311	53.7%	
Beta-blockers	306	52.8%	
Minor diuretics (predominantly hydrochlorothiazide)	273	47.2%	
CCBs	248	42.8%	
Loop diuretics (predominantly Furosemide)	244	42.1%	
Vitamins (predominantly vit. D)	227	39.2%	
Antidepressants + antipsychotics	215	37.1%	
Oral anticoagulants	190	32.8%	
Anxiolytics/hypnotics (Benzodiazepines + Zolpidem)	173	29.9%	
Dietary supplements (predominantly Calcium)	171	29.5%	
Oral antidiabetic drugs	156	26.9%	
Antiasthmatic agents incl. Beta-adrenergics + anticholinergics	134	23.1%	
Opioids	127	21.9%	
Analgesics - Paracetamol	126	21.8%	
Thyroid hormones	114	19.7%	
Corticosteroids	106	18.3%	
NSAIDs + COX-2-inhibitors (Coxibe)	88	15.2%	

*IQR* Interquartile range, *Min* Minimum, *Max* Maximum, *COPD* Chronic obstructive pulmonary disease, *ARBs* Angiotensin II receptor antagonists, *ACE* Angiotensin converting enzyme, *PPIs* Proton pump inhibitors, *CCBs* Calcium channel blockers, *NSAIDs* Non-steroidal anti-inflammatory drugs, *COX* Cyclooxygenase

^a^ If one patient was assuming two active agents from the same drug class, this was counted as one drug class

### PIMs and DDIs

PIMs: 341 drugs (6.1% of all prescribed drugs) were classified as PIM according to the Beers criteria; 266 patients (45.9% of all participating patients) had at least one PIM.

DDIs: 776 severe DDIs were retrieved with 182 *different* drugs being involved in at least one severe DDI. *In total*, 1276 active agents were involved in the 776 DDIs (22.7% of all prescribed drugs); 391 patients (67.5% of all participating patients) had at least one severe DDI (Tables 3 and 4).

**Table 3:** Frequency of potentially inappropriate medications and drug-drug interactions

**Potentially inappropriate drugs according to the Beers-list** [24]				
**Number of PIMs (total)**	Median (IQR)	Mean ± SD^a^	Min	Max
341 (6.1% of all prescribed drugs)	0 (0 – 1)	0.6 ± 0.8	0	4
**n PIMs per patient**	n patients	% of participating patients		
0	313	54.1%		
1	207	35.7%		
≥ 2	59	10.2%		
*TOTAL patients with ≥ 1 Beers-listed drug*	*266*	*45.9%*		
**D or X** ^b^ **drug-drug interactions** [26]				
**Number of DDIs (total)**	Median per patient (IQR)	Min		Max
776	1 (0 – 2)	0		8
**n D or X**^b^ **interactions per patient**	n patients	% of participating patients		
0	188	32.5%		
1	187	32.3%		
2	103	17.8%		
≥ 3	101	17.4%		
*TOTAL patients with ≥ 1 D or X interaction*	*391*	*67.5%*		

*PIMs* Potentially inappropriate drugs, *IQR* Interquartile range, *SD* Standard deviation, *Min* Minimum, *Max* Maximum, *DDIs* Drug-drug interactions

^a^ As the median was 0, in this case also the mean per patient is reported to provide better comprehensibility

^b^ Drug-drug interactions: category D = consider drug modification, category X = avoid combination [26]

**Table 4:** Most common Beers-listed drug classes, DDIs, and drug classes involved in DDIs

**Beers-listed drug classes** [24]	n drugs	% of *n* = 579 patients	**Possible clinical consequences**
Benzodiazepines + Zolpidem	114	19.7%	cognitive impairment, delirium, falls/fractures
NSAIDs + COX-2-inhibitors (Coxibe)	38	6.6%	bleeding, nephrotoxicity
Antiarrhythmics	37	6.4%	toxicity, QT alteration
Alpha-blockers	30	5.2%	hypotension
Diuretics + Spironolacton	29	5.0%	↑ potassium
CCBs	26	4.5%	myocardial ischemia, hypotension
Antithrombotic drugs, mainly Ticlopidine	22	3.8%	toxicity, safer alternatives available
Antidepressants and antipsychotics	20	3.5%	anticholinergic effects, cardio-vascular events, hyponatriaemia
Cardiac glycosides	13	2.2%	toxicity, safer alternatives available
**D**^a^ **or X**^a^ **drug-drug interactions** [26]	n DDIs	% of *n* = 579 patients	**Possible clinical consequences**
Acenocoumarol/Warfarin - Allopurinol	32	5.5%	↑ risk of bleeding
Alendronate - Calcium carbonate	29	5.0%	↓ absorption of alendronate
Amlodipine/Lercanidipine - Simvastatin	27	4.7%	myopathy/ rhabdomyolysis
Levothyroxine - Calcium carbonate	26	4.5%	↓ absorption of levothyroxine
Allopurinol – Ramipril/Lisinopril/Enalapril	21	3.6%	allergic reactions to allopurinol
Bisoprolol - Tamsulosin	18	3.1%	hypotension
Acenocoumarol/Warfarin - Acetylsalicylic acid	16	2.8%	↑ risk of bleeding
Ibuprofen/Diclofenac - Acetylsalicylic acid	15	2.6%	↑ risk of bleeding
Acetylsalicylic acid - Etoricoxib	12	2.1%	↑ toxicity of etoricoxib, bleeding
Clopidogrel - Pantoprazole	11	1.9%	↓ effectiveness of clopidogrel
Bisoprolol - Doxazosin	9	1.6%	hypotension
Ibuprofen - Warfarin	9	1.6%	↑ risk of bleeding
Ibuprofen - Furosemide	8	1.4%	nephrotoxicity
Prednison - Calcium carbonate	7	1.2%	↓ absorption of prednison
**Drug classes involved in D**^a^ **or X**^a^ **DDIs**	n DDIs	% of *n* = 579 patients	
Antithrombotic / anticoagulant drugs	176	30.4%	
Antidepressants / antipsychotics	134	23.1%	
Calcium carbonate	77	13.3%	
Beta-blockers	74	12.8%	
Alpha-blockers	72	12.4%	
Statins	64	11.1%	
NSAIDs + COX-2-inhibitors (Coxibe)	62	10.7%	
ARBs/ACE-inhibitors	61	10.5%	
CCBs	54	9.3%	
Drugs for gout treatment-Allopurinol	48	8.3%	

*NSAIDs* Non-steroidal anti-inflammatory drugs, *COX* Cyclooxygenase, *CCBs* Calcium channel blockers, *DDIs* Drug-drug interactions, *ARBs* Angiotensin II receptor antagonists, *ACE* Angiotensin converting enzyme

^a^ Drug-drug interactions: category D = consider drug modification, category X = avoid combination [26]

### Polypharmacy, PIMs, DDIs and associated factors

The strongest significant associations (p < 0.001) were found between the number of drugs assumed and the number of *severe DDIs* (Spearman’s rho: 0.33) respectively the number of *chronic conditions* (Spearman’s rho: 0.20). Weaker significant associations were found between higher numbers of drugs and a lower *EQ-5D-index/EQ-VAS score* (inverse association; thus, lower health-related quality of life) and a higher *5-GDS score* (thus, depressive status). Similar associations were shown for patients with a use of ≥10 drugs (hyperpolypharmacy). The adjusted associations did not differ substantially from the unadjusted analyses (Table 5).

**Table 5:** Polypharmacy, PIMs and DDIs and associated factors (bold numbers = significant results)

**Polypharmacy in relation to GPs’ and patients’ characteristics**				
**Comparison of median values**	Number of drugs			*p*-value
Patient’s sex	Male	Female		
Median (IQR)	9 (8 – 10)	9 (8 – 11)		0.319 ^a^
GP’s sex	Male	Female		
Median (IQR)	9 (8 – 10)	9 (8 – 11)		0.818 ^a^
Rural / urban area	Rural	Urban		
Median (IQR)	9 (8 – 10)	9 (8 – 10)		0.655 ^a^
**Correlation analysis**	Correlation coefficient			*p*-value
n drugs – patient’s age	0.06			0.146 ^b^
n drugs – n chronic conditions	0.20			**< 0.001** ^b^
n drugs – BMI	0.01			0.817 ^b^
n drugs – 6-CIT score	0.03			0.524 ^b^
n drugs – 5-GDS score (unadjusted)	0.12			**0.003** ^b^
n drugs – 5-GDS score (adjusted)^c^	0.10			**0.017** ^c^
n drugs – EQ-5D index value (unadjusted)	- 0.14			**0.001** ^b^
n drugs – EQ-5D index value (adjusted)^c^	- 0.09			**0.034** ^c^
n drugs – EQ-VAS score (unadjusted)	- 0.14			**0.001** ^b^
n drugs – EQ-VAS score (adjusted)^c^	- 0.11			**0.006** ^c^
**Logistic regression analysis of hyperpolypharmacy** (use of ≥10 drugs)	**Crude analysis**		**Adjusted analysis** (by age, sex, n conditions, 6-CIT)	
	OR	*p*-value	OR	*p*-value
Hyperpolyharmacy – n chronic conditions	1.26	**< 0.001**	1.23	**< 0.001**
Hyperpolyharmacy – 6-CIT score	1.01	0.692	0.99	0.583
Hyperpolyharmacy – EQ-5D index value	0.85	**< 0.001**	0.87	**0.008**
Hyperpolyharmacy – EQ-VAS score	0.86	**0.007**	0.89	**0.035**
Hyperpolyharmacy – 5-GDS score	1.19	**0.021**	1.16	0.065
**PIM use (Beers-listed drugs) in relation to patient’scharacteristics**				
				*p*-value
Patient’s sex	Male	Female		
% of patients with 0 Beers-listed drug	51.7%	55.6%		0.385^d^
% of patients with 1 Beers-listed drug	36.1%	35.5%		
% of patients with ≥2 Beers-listed drugs	12.2%	8.9%		
**Correlation analysis**	Correlation coefficient			*p*-value
n Beers-listed drugs – patient’s age	0.04			0.358 ^b^
n Beers-listed drugs – n chronic conditions	0.05			0.252 ^b^
n Beers-listed drugs – n drugs (unadjusted)	0.07			0.089 ^b^
n Beers-listed drugs – n drugs (adjusted)^c^	0.06			0.156 ^c^
**DDIs in relation to patient’s characteristics**				
				*p*-value
Patient’s sex	Male	Female		
% of patients with 0 D/X drug-drug interactions	30.4%	33.8%		0.374^d^
% of patients with 1 D/X drug-drug interactions	35.7%	30.1%		
% of patients with ≥2 D/X drug-drug interactions	33.9%	36.1%		
**Correlation analysis**	Correlation coefficient			*p*-value
n D/X-DDIs – patient’s age	0.04			0.348 ^b^
n D/X-DDIs – n chronic conditions	0.05			0.220 ^b^
n D/X-DDIs – n drugs (unadjusted)	0.33			**< 0.001** ^b^
n D/X-DDIs – n drugs (adjusted) ^c^	0.33			**< 0.001** ^c^

*PIM* Potentially inappropriate medication, *DDIs* Drug-drug interactions, *GP* General practitioner, *IQR* Interquartile range, *BMI* Body mass index, *6-CIT* 6-Item Cognitive Impairment Test, *5-GDS* 5-Item Geriatric Depression Scale, *EQ-5D* 5-Item questionnaire measuring health-related quality of life, *VAS* Visual analogue scale, *OR* Odds Ratio

^a^ Wilcoxon rank-sum test

^b^ Spearman correlation unadjusted (crude)

^c^ Spearman correlation adjusted by age, sex, number of chronic conditions, cognitive function (6-CIT)

^d^ Fisher’s exact test

The number of drugs did not show a significant association with *patient’s age*, *patients’ sex*, *BMI*, *cognitive function*, *GP’s sex* and geographical *location* of the GP office (rural/urban area). Also, the number of drugs did not correlate with the number of *PIMs*.

In addition, no significant associations were found between the number of PIMs respectively DDIs and *patients’ age*, *patients’ sex* and number of *chronic conditions* (Table 5).

The following *conditions* were associated with higher numbers of drugs: arthrosis, diabetes mellitus, coronary heart disease, COPD (Table 6). Table 6 also shows the association between number of drugs and the most common *drug classes*.

**Table 6:** Polypharmacy and its association with conditions and drug classes (bold numbers = significant results)

**Polypharmacy in relation to the most frequent conditions**	Median (IQR)		*p*-value ^a^
**Chronic conditions**	**n drugs in patients** ***with*** **the condition**	**n drugs in patients** ***without*** **the condition**	
Hypertension	9 (8 – 11)	9 (8 – 10)	0.081
Arthrosis	10 (8 – 11)	9 (8 – 10)	**< 0.001**
Diabetes mellitus II	9 (8 – 11)	9 (8 – 10)	**0.004**
Dyslipidaemia	9 (8 – 11)	9 (8 – 11)	0.901
Atrial fibrillation	9 (8 – 11)	9 (8 – 11)	0.721
Coronary heart disease	10 (8 – 11)	9 (8 – 10)	**0.006**
Osteoporosis	9 (8 – 11)	9 (8 – 11)	0.292
Depression	9 (8 – 11)	9 (8 – 11)	0.051
Benign prostatic hypertrophy	9 (8 – 11)	9 (8 – 11)	0.255
Gastro-oesophageal reflux disease	9 (8 – 10)	9 (8 – 11)	0.885
Chronic heart failure	9 (8 – 11)	9 (8 – 11)	0.443
Chronic obstructive pulmonary disease (COPD)	10 (8 – 12)	9 (8 – 10)	**0.016**
Chronic renal failure	9 (8 – 11)	9 (8 – 11)	0.158
Hyperuricaemia and/or gout	9.5 (9 – 11)	9 (8 – 11)	0.148
Hypothyroidism	9 (8 – 11)	9 (8 – 11)	0.472
Insomnia	9 (8 – 11)	9 (8 – 11)	0.923
Cerebrovascular disease	9 (8 – 11)	9 (8 – 11)	0.986
**Polypharmacy in relation to the most frequent drug classes**	Median (IQR)		*p*-value ^a^
**Drug classes**	**n drugs in patients** ***with*** **the drug class**	**n drugs in patients** ***without*** **the drug class**	
ARBs/ACE-inhibitors	9 (8 – 11)	9 (8 – 10)	0.137
PPIs	9 (8 – 11)	9 (8 – 10)	**< 0.001**
Statins	9 (8 – 11)	9 (8 – 10)	0.257
Platelet-aggregation inhibitors	9 (8 – 11)	9 (8 – 10)	0.056
Beta-blockers	9 (8 – 11)	9 (8 – 10)	0.461
Minor diuretics (predominantly hydrochlorothiazide)	9 (8 – 11)	9 (8 – 11)	0.642
CCBs	10 (8 – 11)	9 (8 – 10)	**0.004**
Loop diuretics (predominantly Furosemide)	10 (9 – 11)	9 (8 – 10)	**< 0.001**
Vitamins (predominantly vit. D)	10 (8 – 11)	9 (8 – 10)	**< 0.001**
Antidepressants	10 (9 – 12)	9 (8 – 10)	**< 0.001**
Oral anticoagulants	9 (8 – 11)	9 (8 – 11)	0.620
Anxiolytics/hypnotics (Benzodiazepines + Zolpidem)	10 (8 – 11)	9 (8 – 10)	**< 0.001**
Dietary supplements (predominantly Calcium)	10 (9 – 11)	9 (8 – 10)	**< 0.001**
Oral antidiabetic drugs	9.5 (8 – 11)	9 (8 – 10)	**0.018**
Antiasthmatic agents, beta-adrenergics, anticholinergics	10 (9 – 12)	9 (8 – 10)	**< 0.001**
Opioids	10 (9 – 12)	9 (8 – 10)	**< 0.001**
Analgesics - Paracetamol	9 (8 – 11)	9 (8 – 11)	0.385
Thyroid hormones	10 (8 – 11)	9 (8 – 10)	**0.009**
Corticosteroids	10 (9 – 12)	9 (8 – 10)	**< 0.001**
NSAIDs + COX-2-inhibitors (Coxibe)	9 (8 – 10)	9 (8 – 11)	0.716

*IQR* Interquartile range, *COPD* Chronic obstructive pulmonary disease, *ARBs* Angiotensin II receptor antagonists, *ACE* Angiotensin converting enzyme, *PPIs* Proton pump inhibitors, *CCBs* Calcium channel blockers, *NSAIDs* Non-steroidal anti-inflammatory drugs, *COX* Cyclooxygenase

^a^ Wilcoxon rank-sum test

## Discussion

A summary of the main results of the study is provided in Table 7.

**Table 7:** Summary of the main study findings

**Drug use**	13.4% of patients aged ≥75 years were prescribed ≥8 drugs
	Median drug use among the included patients: 9 drugs
	Most commonly used drug classes: Angiotensin II receptor antagonists / ACE-inhibitors, protonic pump inhibitors, statins, platelet-aggregation inhibitors, beta-blockers
**Chronic conditions**	64.9% of the participating patients had ≥5 diagnoses of chronic conditions
	Most frequent diagnoses: Arterial hypertension, arthrosis, diabetes mellitus II, dyslipidaemia, atrial fibrillation
**PIMs**	45.9% of patients were treated with at least one PIM according to the 2012 Beers criteria
	Most common PIMs: Benzodiazepines and Zolpidem, NSAIDs / COX-2-inhibitors (Coxibe), antiarrhythmics
**DDIs**	67.5% of patients were exposed to at least one major DDI
	Drug classes most frequently involved in major DDIs: antithrombotic / anticoagulant drugs, antidepressants / antipsychotics, calcium carbonate
**Polypharmacy and associated factors**	Significant correlations (*p* < 0.05): • number of drugs – number of major DDIs (Spearman’s rho: 0.33) • number of drugs – number of chronic conditions (Spearman’s rho: 0.20) • number of drugs – reduced affective status (Spearman’s rho: 0.12) • number of drugs – reduced QoL / health status (Spearman’s rho: −0.14)
	Conditions associated with higher numbers of drugs: Arthrosis, diabetes mellitus II, coronary heart disease, COPD

*ACE* Angiotensin converting enzyme, *PIMs* Potentially inappropriate medications, *NSAIDs* Non-steroidal anti-inflammatory drugs, *COX* Cyclooxygenase, *DDIs* Drug-drug interactions, *QoL* Quality of life, *COPD* Chronic obstructive pulmonary disease

### Drug use, prevalence of polypharmacy and most common drug classes

The *number of drugs* used within our study cohort was high: on average, patients took nine active agents per day. A similar degree of drug consumption was found also in other studies with a comparable setting [31]. Yet, also studies including not only patients on polypharmacy detected high average drug numbers among older adults (eight prescriptions) [32, 33].

Also the *prevalence of polypharmacy* in our study was considerable: 13.4% of general practice patients aged 75+ were on therapy with ≥8 drugs. This rate, although being not always fully comparable to previous studies due to different definitions of polypharmacy, can however be considered to lie within the range reported by others. A northern Italian study found that 48% of general practice patients aged 65+ were using 5-9 drugs (polypharmacy) and 10% used ≥10 drugs (hyperpolypharmacy) [34]. Similar results were reported by a Swedish cohort study [35] and by an Italian population study [11]. The rate of patients using ≥10 drugs in our sample (43%, Table 2) seems much higher; however, as we included only patients taking ≥8 drugs and were not able to stratify the use of drugs within the *whole* older-aged population, this number is not comparable to the hyperpolypharmacy rates detected by others.

A study that used the same cut-off as in our cohort (≥8 drugs) detected a sharply higher prevalence of polypharmacy (50%), however, the study had been conducted in the inpatient setting [36]. An Austrian study assessing hospital-admitted patients aged 75+ and on therapy with ≥7 drugs found an even higher prevalence of polypharmacy of 58% [37].

In general, as studies often differ in terms of definition of polypharmacy, investigated age groups and settings, comparability between studies is limited. Moreover, results from studies with comparable cut-offs also differ largely. A northern Italian study using administrative databases of the local health authorities detected a rate of polypharmacy (≥5 drugs) of 17% in patients aged 65+ [38] while an Italian population study including all drugs reimbursed by the National Healthcare System (NHS) showed 49% of people aged 65+ to be on therapy with 5-9 drugs and 11% with ≥10 drugs [11].

These notable differences within the same country suggest that polypharmacy rates depend not only on setting but may also vary according to local-regional differences and the used data sources. Moreover, differences between polypharmacy rates can even be considerable between single physicians: In our study, the prevalence of polypharmacy ranged from 5 to 37% among the participating GPs. A similar phenomenon was described in other studies [39]. Factors that determine variations in prescribing rates are not well understood; demographic characteristics of patients and GPs themselves may play a role as well as the GP-patient relationship and/or GP-related factors (different prescribing patterns based on training, practice organisation etc.) [39].

The most often used *drug classes* in our study were in accordance with previous investigations [33, 40]. Concordantly to the most prevalent chronic condition hypertension and in consistence with the respective recommended first-line treatment [41] the most frequently prescribed drug classes were ARBs/ACE-inhibitors (83%) with ARBs being slightly more frequently used than ACE-inhibitors (Table 2). ACE-inhibitors are recommended to be used prior to ARBs due to their lower costs and similar benefit [41] except in case of contraindications.

About half of the investigated patients received PPIs, statins, antithrombotic agents, beta-blockers and/or minor diuretics. 40-30% were treated with CCBs, loop diuretics, vitamin D, antidepressants, oral anticoagulants, benzodiazepines and/or calcium.

The large European multicentre trial PRIMA-eDS which included primary care patients from UK, Austria, Italy and Germany and used similar inclusion criteria (patients aged 75+ and taking ≥8 drugs) reported the same top five drug classes. As in our cohort, ACE-inhibitors/ARBs were the most frequent therapeutic subgroup (80%), followed by statins which were prescribed more often than in our sample (64% vs. 55%); the prescription rates of PPIs, beta-blockers and antithrombotic agents were approximately similar to our study [19].

Other studies reported statins to be the most used drug class in older patients [42, 43] respectively diuretics [37] respectively beta-blockers [31]. In the latter study, NSAIDs (28%) were prescribed considerably more often than in our cohort (15%), however, as we did not include OTC-drugs and some (however only low-dose) NSAIDs are available without prescription in Italy, the use of NSAIDs in our study could slightly have been underestimated.

The use of dietary supplements within our sample was high: 39% of patients were treated with vitamin D and 30% with calcium. A US population study found even 64% of older persons to regularly use dietary supplements [43]. In general, evidence regarding dietary supplements is not convincing; however, some research suggested possible beneficial effects of vitamin D on falls and cognitive function in older adults which may have contributed to its frequent and increasing use [43].

### Chronic conditions

The included patients were multimorbid with on average five chronic conditions per patient. Nearly two thirds (65%) of the included patients were diagnosed with ≥5 chronic conditions.

Arterial hypertension was by far the most frequent diagnosis (85% of patients), followed by arthrosis, diabetes mellitus type 2, dyslipidaemia, atrial fibrillation and coronary heart disease. About one fifth of the investigated patients suffered from depression. The ranking of the most prevalent conditions was comparable to other polypharmacy studies [19, 34].

Population studies of older patients with similar mean age [2] found a considerably higher prevalence of dementia (21%) compared to our sample. This may be related to (a) a true lower rate of polypharmacy among dementia patients which was confirmed also by other research [44] due to lower life expectancy and less benefit of multiple pharmacological treatment, or to (b) a possible under-representation of dementia in our study cohort, possibly also due to the fact that severe cognitive dysfunction was an exclusion criterium (only four of 579 patients were explicitly diagnosed with dementia, Table 2).

### Potentially inappropriate medications

Nearly half of patients (46%) in our cohort were treated with at least one PIM, 10% received ≥2 Beers-listed drugs. On average, one out of two patients received PIMs. The most frequent PIM classes were benzodiazepines/hypnotics (20% of patients) and NSAIDs (7%), followed by antiarrhythmics and alpha-blockers. Similar distributions of PIM classes were detected by other studies [34, 37, 45] while PIM prevalences varied notably in previous studies on community-dwelling older persons (12- 69%) [32, 45]. A study using the 2015 Beers criteria reported 46% of older-aged primary care patients to be treated with ≥2 Beers-listed drugs [45]; this is more than fourfold higher than in our cohort. The most frequently used Beers-listed drug classes were PPIs (46% of patients), and, as in our sample, NSAIDs and benzodiazepines. PPIs were not detected as PIMs in our study since we used the 2012 Beers criteria (in the 2015 and later versions of the Beers criteria, PPIs were added as potentially inappropriate medications when used beyond eight weeks without clear indication, as evidence supported an association between long-term use of PPIs and *Clostridium difficile* infection and bone loss) [46]; however, it is noteworthy that only one fifth of patients in our cohort were diagnosed with gastro-oesophageal pathologies while 55% received PPIs. This leads to the assumption that PPIs were probably inappropriately used also in a notable percentage of patients within our cohort. The fact that the Italian Medicines Agency (AIFA, Agenzia Italiana del Farmaco) stipulates a prescription of PPIs at the charge of the National Health Service in case of antiaggregant therapy with acetylsalicylic acid independently from gastrointestinal risk [47] had probably a relevant impact on the PPI prescription rates in our sample. The Italian multicenter prospective CRIME study found PPIs to be inappropriately prescribed in 30% of hospital-discharged older patients, however, also under-prescription of PPIs was found in 11% of patients in this cohort, particularly in older patients with higher numbers of comorbidities and drugs [48].

Benzodiazepines are frequently used in primary care although their risk of falls/fractures and cognitive impairment is well-known [49]. They were among the most common drug classes also in our cohort (30%). Although we did not assess the specific indication for each administered drug, according to the most prevalent diagnoses it may be assumed that benzodiazepines were prescribed mostly for insomnia (10% of patients) and/or depression (21%). According to a German qualitative study [49], benzodiazepines are frequently prescribed also in case of insistent patient’s demands in absence of serious or clear indications. Thus, GPs are faced with the challenge to address both medical recommendations and patient’s expectations and to do so within a reasonable timeframe. On the other hand, a UK study showed that patients are often not adequately informed about the risks of benzodiazepines and that patients’ willingness to attempt therapy withdrawal is given [50]. Therefore, increased patient information is required which can support the GPs’ efforts regarding avoidance or withdrawal of inappropriate benzodiazepine prescriptions; this remains, however, difficult in case of drug dependency after long-term benzodiazepine use.

NSAIDs were prescribed to 15% of patients in our sample; in half of these cases, they were rated as inappropriate. This seems to be lower when compared to other studies [45]; however, NSAIDs have a well-known, high potential for DDIs and ADEs in older persons especially in case of comorbidities and concomitant corticosteroid or antiplatelet/anticoagulant medication and have also shown to be associated with major cardiovascular events independently from baseline cardiovascular risk [42]. For daily practice, it is recommended that NSAIDs should be possibly avoided in older patients [42]; however, complete avoidance is difficult, not least because of the frequent arthrosis-related disorders in this age group. Thus, thorough careful risk-benefit consideration is required.

Overall, studies confirm the harmful impact of PIM use. A recent systematic review showed PIM use to be associated with increased mortality risk in older adults [51]. Therefore, and as PIMs were prescribed to nearly half of patients in our cohort, physicians’ awareness towards more restrictive PIM use should be increased. Yet, in some individual cases, a valid indication for the use of a Beers-listed drug might be given, e.g. amiodarone in therapy-resistant arrhythmias [37].

### Drug-drug interactions

About two thirds (68%) of patients in our sample were exposed to at least one major interaction and more than one third had ≥2 major interactions. 17% were exposed to ≥3 major interactions. On average, every patient had one major DDI. These numbers show a high prevalence of DDIs and were largely confirmed also by other research [31, 37].

Other studies found higher average frequencies of DDIs in older-aged primary care patients on polypharmacy (1.5 DDIs per patient) respectively in a hospital setting (2.6 DDIs per patient) [2, 38]. In our sample, the most common EHR used by the participating GPs integrated a DDI-checking programme which alerted the physicians in case of clinically relevant DDIs at any drug prescription; this could have led a priori to a reduction of DDIs in our cohort.

In general, comparability of DDIs between studies is limited due to incongruent grading tools, different ways of reporting (all retrieved DDIs vs. ‘clinically relevant’/ ‘severe’/ ‘high-risk’ DDIs) and different drug availabilities and prescribing patterns across countries and settings [38]. However, the prevalence of DDIs in our cohort is still alarming despite EHR software use: a multivariate analysis found a significant association between exposition to ≥2 severe DDIs and an increased risk of 3-months-mortality [52].

In consistence with other studies [2], antithrombotic and anticoagulant medications were the most frequently interacting drugs (30% of patients) followed by antidepressant and antipsychotic medications (23%), calcium carbonate and beta-blockers (13%). Regarding antithrombotic and anticoagulant drugs, which are characterised by a narrow therapeutic window and a high potential for DDIs [40], the most frequent interactions were with allopurinol, acetylsalicylic acid and NSAIDs. All these DDIs carry an increased risk of haemorrhagic complications [26]. Also calcium carbonate was commonly involved in major DDIs. Calcium interferes with the absorption of other active agents thus lowering their plasma level; this can, however, be avoided by separating the administration and/or dose adjustment [26].

The most common DDIs in our sample implicated commonly used drugs. These findings are hardly surprising; however, they suggest that GPs in daily practice should give more attention to combinations of frequently used drugs which may lead to avoidable ADEs. Thus, careful therapy monitoring in older patients with multiple conditions should be conducted regularly. On the other hand, also potentially severe DDIs can sometimes be accepted if the expected benefit of a combination therapy (e.g. double antiplatelet/anticoagulant therapy) outweighs the risk; this requires an appropriate and continuous patient monitoring and counseling [38].

### Polypharmacy and associated factors

As in other studies [19, 34, 35, 40], *multimorbidity* was an important factor associated with polypharmacy (independently from patients’ age and gender). Especially diabetes, coronary heart disease, COPD and arthrosis were associated with high numbers of drugs in our sample. Other authors reported cardiometabolic diseases and COPD [9, 44] respectively hypertension [37, 53], depression and pain [37, 53, 54] as predictors of polypharmacy.

Most of these conditions are highly prevalent in older-aged persons and their evidence-based treatment often requires the administration of several drugs. Thus, multiple drug use may sometimes be unavoidable and polymedication is not always synonymous with an inappropriate overuse of drugs. E.g., a study in German nursing homes showed a postinterventional increase of medication appropriateness despite of unchanged numbers of drugs [6].

As in other research [37], we detected no association between polypharmacy and *patients’ age*. Study findings in this regard are contradicting. Some research detected almost linear associations with the number of drugs remaining high among the oldest old [35, 40, 54, 55]. This was confirmed also by the Italian EPIFARM study [56]. In contrast, the Italian CRIME study found no variation of drug numbers across age groups [57]; this study included, however, inpatients of geriatric and internal medicine acute care wards. Other European studies found the age of 85+ to be a protective factor against excessive polypharmacy [19, 58] and claimed as possible explanations (1) a real lower drug use in this age group because of reduced life expectancy, or (2) the possibility that patients exposed to excessive polypharmacy died already before reaching the very old age [19]. Also, reduced patients’ compliance could have played a role.

*Patients’ sex* was not linked to polypharmacy in our sample. Some studies found male sex [40, 58], other studies reported female sex [37, 54, 55] to be associated with polypharmacy. Our results as well as previous findings [19] suggest that polypharmacy is an equivalent phenomenon in both male and female patients and should therefore be observed independently from patients’ sex.

No associations between *cognitive status* and polypharmacy were noted in our sample as well as in previous research [37], however, polypharmacy showed weak but significant associations with an impaired *affective status* and lower health-related *quality of life* and *self-reported health status*. The latter findings were confirmed by other studies [19, 54]. Yet, our analyses do not allow an affirmation regarding causality: polypharmacy itself may impair quality of life and affective status (e.g. due to ADEs), or an impaired functional status (e.g. due to multimorbidity) may entail the risk for polypharmacy. We assume that both explanatory models may play a role. Consequently, when prescribing drug therapies, the GP has to pay particular attention to patients with impaired health and affective status.

In contrast to other studies [37], *PIM use* in our sample was not related to the number of drugs. Evidence regarding the association between PIM use and patients’ age, gender and multimorbidity is contradicting [32, 45, 59]. E.g., a higher PIM use was found in patients with higher number of chronic conditions [32]; another study confirmed this only for males [59].

Polypharmacy in our cohort was significantly associated with the *number of major DDIs*. This was the strongest association (although it has still to be considered as weak) and has been confirmed by previous studies [37, 55, 60]. Evidence regarding associations between DDIs and other patient-related factors (which were missing in our cohort) is inconsistent; some research detected higher DDI prevalences in patients with older age [55], male sex [43, 55] respectively multimorbidity [60].

Most of the commonly used *drug classes* in our sample were significantly more prevalent in patients with higher overall numbers of drugs (Table 6). This phenomenon, which was also observed in other studies [37], largely reflects the most frequent diagnoses within our cohort. It is, however, noteworthy that of the five most common drug classes (ACE-inhibitors/ARBs, PPIs, statins, platelet-aggregation inhibitors, beta-blockers) only PPIs were directly associated with higher overall numbers of drugs. PPIs are likely to be prescribed in patients with higher drug intake for ‘stomach protection’ and- as our results confirm- sometimes without clear indication (see above). Thus, PPI use should be questioned and stopped more rigorously.

### Strengths and limitations

A strength of our study is that we assessed not only polypharmacy in combination with a range of demographic and clinical characteristics, but also medication appropriateness and DDIs.

In contrast to other studies [38, 40] we were able to provide information not only about drugs reimbursed by the NHS, but we included all prescribed drugs according to the physicians’ EHRs. However, OTC-medications were not assessed because the electronic data extraction was possible only for *prescribed* drugs which were the only drugs recorded in the EHRs. OTC-drugs could have been additionally collected by questioning the participating patients. However, older-aged patients not always remember all drugs they are taking and brown bag medication reviews with each patient were not feasible within the logistic constraints of the study. Thus, a reliable and complete determination of OTC-drugs was not possible and was therefore a priori excluded.

A German study showed that the prevalence of polypharmacy doubled when OTC-medications were included [53]. Thus, medication use in our study might have been underestimated; however, as in Italy most continuously taken drugs are only available on prescription (except e.g. some low-dose NSAIDs or vitamins), the exclusion of OTC-drugs should not have led to a substantial bias.

Also, new drugs prescribed by specialists and not yet appearing in the GPs’ EHRs could have been missed; however, we assume that this does not play a relevant role in our sample as in Italy repeated prescriptions of chronic drug therapies are usually conducted by the GPs.

Our analysis was limited to regularly used drugs and we excluded PRN-medications. Thus, drugs used for acute conditions which might have interacted with other medications and conditions could have been overlooked as well.

We used the Beers criteria instead of European tools which might have been more adapted to the local market [61]; further limitations of the Beers criteria are e.g. missing inclusion of duplicate prescriptions or underuse of indicated drugs [61] as well as not-considering of concomitant conditions and possible drug-disease interactions [62]. Nevertheless, the Beers criteria are widely used which facilitates international comparability; moreover, at the moment of the start of our study, the Beers criteria were more established in Italy compared to other European tools (e.g. STOPP/START criteria) [63, 64] and the last update available for the Italian version of the Beers criteria was published in 2012, recently before the development of the PRIMA study protocol, and was adapted to the Italian market and availabilities of drugs.

In general, information for the GPs regarding patients’ real drug consumption needs to be improved especially in case of multiple prescribers and use of OTC-drugs; hereby, electronic medical records merging information about all medical consultations and drug purchases can provide useful support.

We only enrolled patients aged 75 years or more. Thus, the comparability with other studies which often included younger persons is limited. However, this is also a strength of our study as the older age groups are less studied up to now although being the most vulnerable cohort of patients [56].

A further strength is the broad inclusion of general practice patients instead of a pre-selected cohort of patients admitted to specific hospital wards. The GP sample as well as the patient sample was consecutively recruited to reduce the risk of selection bias, however, the GP sample was small and can therefore not be considered as representative. The patient sample was somewhat selected as we enrolled only community-living patients who visited the GP office. More motivated GPs were probably more willing to participate in the study and therefore a selection bias cannot be excluded. Generalisability is further limited by the fact that our results are derived from a northern Italian region and epidemiology of polypharmacy might differ in other populations. However, as stated above, many studies in other European countries have shown similar results which leads to the assumption that our results might be comparable to other national contexts and that the implications of the study can therefore be applicable also to other European countries. As drug prescription patterns vary depending on the respective healthcare system, the results of our study might be more comparable to countries with a similar healthcare system as in Italy (NHS-based).

We a priori only included patients with major polypharmacy and the cut-off applied in our study (≥8 drugs) is not very commonly used; thus, comparability with other studies is limited.

We did not assess other covariates with potential independent impact on drug consumption (beyond their possible relationship with patients’ age), e.g. frailty or educational level.

Due to the cross-sectional design of this study, no conclusions on causal relationships could be drawn. The publication of the results of the RCT from which this cross-sectional analysis is derived will provide information on causality and outcomes.

## Conclusions

Polypharmacy in our sample was associated with the number of chronic conditions, with a higher number of major DDIs, and with lowered patients’ affective status and quality of life. Patients’ age, patients’ sex as well as GPs’ age, GPs’ sex and geographical location did not entail a higher risk of polypharmacy. Also, polypharmacy was not related to patients’ cognitive status, BMI or to the use of PIMs. Although polypharmacy is not synonymous with inappropriate treatment, it is well-known that it increases the risk of adverse events. Our results indicate that GPs should particularly keep their patients with multiple conditions, reduced health and affective status under surveillance, especially those diagnosed with arthrosis, diabetes, coronary heart disease and COPD, independently from patients’ age or sex. Regular medication reviews for these patients by GPs with or without consideration of a multidisciplinary approach (e.g. support of a clinical pharmacist) and/or electronic decision support tool should be implemented as routine measure in general practice care with the prerequisite that the GPs are provided with appropriate time resources and technical/financial support.

We did not identify individual therapeutic subgroups which might be particularly considered as risk drug classes for polypharmacy. However, some drug classes were highly represented among the prescriptions rated as PIMs: especially benzodiazepines should be carefully considered regarding indication, benefit and potential risk. Moreover, patients with antithrombotic/anticoagulant drugs, antidepressants and/or antipsychotics are prone to major DDIs and should be adequately monitored.

In daily practice, the questions supported by our findings should be (a) if the used drugs are necessary and appropriate, (b) if the benefit of a drug outweighs its risk for this specific patient with his/her characteristics, comorbidities and concomitantly used drugs, (c) if the altogether of drugs minimises the risk for undesired harmful consequences, (d) if the applied drug regimen serves to maintain the best possible quality of life, and not least (e) if it is practical and acceptable for the patient.

## Data Availability

The datasets used during the current study are available from the corresponding author on reasonable request.

## Abbreviations

ACE: Angiotensin converting enzyme
ADE: Adverse drug event
ARBs: Angiotensin II receptor antagonists
BMI: Body mass index
CCBs: Calcium channel blockers
6-CIT: 6-Item Cognitive Impairment Test
COPD: Chronic obstructive pulmonary disease
COX: Cyclooxygenase
DDIs: Drug-drug interactions
EHR(s): Electronic health records
EQ-5D-5L and EQ-VAS: 5-level EQ-5D version (5-Item questionnaire and visual analogue scale measuring health-related quality of life and self-reported health status)
5-GDS: 5-Item Geriatric Depression Scale
GP: General practitioner
ICD-9: International Classification of Diseases, Ninth Revision
IQR: Inter-Quartile-Range
NHS: National Healthcare System
NSAIDs: Non-steroidal anti-inflammatory drugs
OTC-drugs: Over the counter medications (available without prescription)
PIM: Potentially inappropriate medication
PPIs: Proton pump inhibitors
PRIMA: Polypharmacy in chronic diseases - Reduction of Inappropriate Medication and Adverse drug events in older populations (study acronym)
PRN-drugs: Pro re nata (as needed) medication
QoL: Quality of life
RCT: Randomized controlled trial
SD: Standard deviation

## Footnotes

1 In contrast to the European multicenter trial PRIMA-eDS [19], the here presented PRIMA study was limited to a northern Italian region and applied medication reviews provided by three experts (a specialist in internal medicine, a clinical pharmacist and an EbM expert) as intervention. The data generated by the independent Italian PRIMA study were *not* part of the PRIMA-eDS trial.

2 The EQ-5D user guide recommends using value sets of a country that most closely approximates to the country of interest in case of absence of a country-specific value set [29].
